# A Deubiquitinating Enzyme Ubp14 Is Required for Development, Stress Response, Nutrient Utilization, and Pathogenesis of *Magnaporthe oryzae*

**DOI:** 10.3389/fmicb.2018.00769

**Published:** 2018-04-18

**Authors:** Zhao Wang, Hong Zhang, Caiyun Liu, Junjie Xing, Xiao-Lin Chen

**Affiliations:** ^1^The Provincial Key Lab of Plant Pathology of Hubei Province, College of Plant Science and Technology, Huazhong Agricultural University, Wuhan, China; ^2^State Key Laboratory of Hybrid Rice, Hunan Hybrid Rice Research Center, Changsha, China

**Keywords:** deubiquitinating enzyme, ubiquitination, carbon source utilization, pathogenesis, protein degradation

## Abstract

Ubiquitination is an essential protein modification in eukaryotic cells, which is reversible. Deubiquitinating enzymes (DUBs) catalyze deubiquitination process to reverse ubiquitination, maintain ubiquitin homeostasis or promote protein degradation by recycling ubiquitins. In order to investigate effects of deubiquitination process in plant pathogenic fungus *Magnaporthe oryzae*, we generated deletion mutants of *MoUBP14*. Ortholog of MoUbp14 was reported to play general roles in ubiquitin-mediated protein degradation in *Saccharomyces cerevisiae*. The Δ*Moubp14* mutant lost its pathogenicity and was severely reduced in mycelial growth, sporulation, carbon source utilization, and increased in sensitivity to distinct stresses. The mutant was blocked in penetration, which could due to defect in turgor generation. It is also blocked in invasive growth, which could due to reduction in stress tolerance and nutrient utilization. Deletion of *UBP14* also led to accumulation of free polyubiquitin chains. Pulldown assay identified some proteins related to carbohydrate metabolism and stress response may putatively interact with MoUbp14, including two key rate-limiting enzymes of gluconeogenesis, MoFbp1 and MoPck1. These two proteins were degraded when the glucose was supplied to *M. oryzae* grown in low glucose media for a short period of time (∼12 h), and this process required MoUbp14. In summary, pleiotropic phenotypes of the deletion mutants indicated that MoUbp14 is required for different developments and pathogenicity of *M. oryzae*.

## Introduction

Ubiquitination is an important post-translational modification, which usually marks cellular proteins for degradation through the 26S proteasome in eukaryotic cells (Collins and Goldberg, 2017). The ubiquitin molecules are mostly linked to one another between the C terminus of one ubiquitin and lysine 48 of the next ubiquitin. During linking process, a ubiquitin activating enzyme (E1) is required to activate the 76 amino acids ubiquitin. The activated ubiquitin is then transferred to an ubiquitin conjugating enzyme (E2) and modifies target proteins by assistance of the ubiquitin ligase (E3) (Callis, 2014). Mostly, proteins bearing four or more ubiquitin chains are recognized and degraded by the 26S proteasome complex (Collins and Goldberg, 2017). Attachment of ubiquitin to cellular proteins is important for regulating distinct cellular processes, such as DNA repair, cell-cycle progression, stress responses, signal transduction, endocytosis, programmed cell death, etc (Dikic et al., 2009).

Protein ubiquitination is a reversible event, and the removal of ubiquitins from proteins is also essential. In fact, the ubiquitin monomers should be kept in homeostasis, which is mainly determined by processing ubiquitin precursors or deubiquiting ubiquitins from substrates (Komander et al., 2009). Deubiquitination is directed by de-ubiquitinating enzymes (DUBs), a special group of thiol proteases, which are of high specificity for removing of the ubiquitin moiety. DUBs play key roles in several aspects of the ubiquitin-dependent processes (Komander et al., 2009). Firstly, DUBs can release the ubiquitin moieties linked to polyubiquitin and ubiquitin extension proteins, which are required for the homeostasis of the 76-amino acid ubiquitin monomer pools. Secondly, DUBs can reverse ubiquitination processes. For example, deubiquitination of Lys48-linked multi-ubiquitinated proteins by DUBs could prevent the proteins from degrading by the 26S proteasome. Thirdly, DUBs can recycle ubiquitins for reutilization. This role can be achieved by removing small peptide fragments that remain bound to the ubiquitins during ubiquitin-mediated protein degradation, and also by releasing the free ubiquitin monomers through disassembling the multi-ubiquitin chain (Komander et al., 2009). Different attachment determines distinct functions of ubiquitination. The most widespread and best characterized ubiquitination is the attachment of a ubiquitin chain linked with Lys48 residue. This type ubiquitination will lead to degradation of the target proteins via the 26S proteasome. While other types ubiquitination, including Lys6, Lys11, Lys27, Lys29, Lys33, and Lys63-linkages, may result in non-proteolytic processes such as transcription, chromatin structure formation, vesicular trafficking, and so on (Ikeda and Dikic, 2008).

The ubiquitin-specific protease (UBP) is a large family of DUBs, which contains six conserved regions, and the Cys and His boxes are essential for catalysis (Hu et al., 2002). In *Saccharomyces cerevisiae*, there are 16 *UBP* genes (Wilkinson, 1997), while in *Arabidopsis thaliana*, there are 27 *UBP* genes (Liu Y. et al., 2008). Studies in model fungi such as *S. cerevisiae* have revealed that UBPs are involved in nutrient utilization, energy metabolism, sexual reproduction, and stress responses (Kahana, 2001; Enyenihi and Saunders, 2003; Dudley et al., 2005; Auesukaree et al., 2009). For example, Doa4 is important for carbon utilization, Ubp10 is required for nitrogen utilization (Enyenihi and Saunders, 2003), and Ubp15 plays important roles in growth and stress response (Yoshikawa et al., 2011). Ubp14 is the best known DUB, which plays general roles in proteasome-mediated proteolysis in *S. cerevisiae* and human (through USP5 or isopeptidase T ortholog) (Hadari et al., 1992; Amerik et al., 1997; Eisele et al., 2006). In *S. cerevisiae*, Ubp14 functions in disassembling free polyubiquitin chains which are liberated from ubiquitinated proteins before their proteasomal degradation (Falquet et al., 1995; Wilkinson et al., 1995). Deletion of the *S. cerevisiae UBP14* leads to accumulation of free polyubiquitin chains and inhibition of proteasomal degradation (Amerik et al., 1997). Similar accumulation of free polyubiquitin chains was also found in deletion mutants of human *USP5* and *Drosophila DmUsp5* genes (Dayal et al., 2009; Kovács et al., 2015). These studies reveal that Ubp14 functions as a ubiquitin recycler to remove the free ubiquitin chains, and consequently helps in supplying sufficiently free ubiquitins to maintain a monoubiquitin pool. Deletion of *S. cerevisiae UBP14* also leads to defect in sporulation and was hypersensitive to the arginine analog canavanine (CAN) (Amerik et al., 1997). In human, *USP5* is found to be required for DNA double-strand repair in HeLa cells (Nakajima et al., 2014). In *Drosophila*, *DmUsp5* is required for maintenance of the cell survival and normal development, and loss of the DmUsp5 function results in late larval lethality and apoptosis induction (Kovács et al., 2015). However, whether UBPs, especially Ubp14, are involved in pathogenesis of the plant pathogenic fungi is still unknown.

*Magnaporthe oryzae* is a hemi-biotrophic ascomycete fungus, which is a serious threat to rice production, and it has become a model plant fungal pathogen (Wilson and Talbot, 2009; Yan and Talbot, 2016). This fungus can form a specialized appressorium on the host surface, and generate turgor pressure for penetration (de Jong et al., 1997; Dixon et al., 1999; Thines et al., 2000). During appressorium maturation, the lipid body and glycogen are mobilized for nutrient and energy consumption (Weber et al., 2001; Wang et al., 2003). Once intruding into host cells, the fungus forms filamentous primary hypha and bulbous secondary invasive hyphae (IH) to establish biotrophic colonization (Kankanala et al., 2007). At last, the fungus converts into necrotrophic growth for conidia production. During different infection stages, the fungus should elaborately coordinate its metabolic processes for nutrient assimilation (Fernandez and Wilson, 2014). In this study, we use *M. oryzae* as a model to investigate roles of the deubiquitinating enzyme Ubp14 in the plant pathogenic fungi. We found that deletion of *M. oryzae UBP14* resulted in loss of virulence, as well as pleiotropic phenotypic defects, including slower in colony growth, reduction in conidiation, increase in sensitivity to stresses, and reduction in carbon sources utilization. In addition, we also found that deletion of MoUbp14 affected degradation of Fbp1 and Pck1, two key rate-limiting enzymes in the gluconeogenesis. Our work illustrates the importance of Ubp14 in the plant pathogenic fungi.

## Materials and Methods

### Strains and Culture Conditions

The *M. oryzae* strain P131 was used as a wild type (Chen et al., 2014). All of the wild-type strain and transformants used in this study were grown on Oatmeal Tomato Agar (OTA) plates at 28°C. To extract genomic DNA, RNA, protein, and isolate protoplasts, mycelia were incubated in liquid CM cultures (180 rpm) at 28°C for 36 h. Colony growth and conidiation were performed as described previously (Chen et al., 2014). Conidia harvested from 7-day-old OTA cultures were used for testing virulence and observing infection process.

To test stress sensitivities, colony diameter of different strains were measured at 5 days post inoculation (dpi) on CM plates added with 0.2 mg ml^-1^ Congo Red (CR), 0.1 mg/ml Calcofluor White (CFW), 0.005% Sodium dodecyl sulfate (SDS), 0.5 M NaCl, 10 mM H_2_O_2_, or buffered at pH 8.0 with phosphate buffer. The diameters of the colonies were recorded and used for calculating the growth reduction rates (Chen et al., 2014).

To test carbon sources utilization, colony diameter of different strains were measured at 5 dpi on MM agar plates amended with 1% glucose, 5 mM sodium acetate, ethanol, or glycerol as sole carbon source. The diameters of the colonies were recorded for the calculation of the growth reduction rates (Kong et al., 2012).

### Gene Disruption and Complementation

To generate gene’s replacement construct, 1.5-kb upstream and downstream of the gene’s flanking sequences were amplified from the genomic DNA of the wild-type strain. Both flanking sequences of *MoUBP14* were cloned into pKNH as the deletion vector, and then transformed into protoplasts of the wild type (Chen et al., 2014). For complementation, *MoUBP14* gene containing 1.5 kb promoter region and 0.5 kb terminator region was amplified and cloned into pKN (Chen et al., 2014). The resulting constructs were transformed into the Δ*Moubp14* mutant. CM plates supplemented with 250 μg ml^-1^ hygromycin B (Roche, United States) was used to select deletion transformants, or supplemented with 400 μg ml^-1^ neomycin (Amresco, United States) to select complementation transformants. The deletion transformants were verified by PCR and confirmed by Southern blot.

### Subcellular Localization

The *eGFP:MoUBP14* fusion vector was generated by an overlap method. A PCR product including 1.5 kb of the promoter region, eGFP gene without termination codon, and ORF region of the *MoUBP14* gene was sequentially ligated by an overlap-PCR method. Then this product was cloned into pKN. The resulting construct pKNG-UBP14 was transformed into the Δ*Moubp14* mutant. Subsequent complementary strains were used to observe GFP fluorescence at different developmental stages under a confocal microscope Leica TCS SP8 (Leica Microsystems, Germany).

### Virulence Test and Infection Process Observation

To test virulence of different fungal strains, 1-week-old barley leaves (*Hordeum vulgare* cv. E9) and 1-month-old rice seedlings (*Oryza sativa* cv. LTH) were sprayed with conidia suspensions (5 × 10^4^ conidia ml^-1^) in 0.025% Tween 20. The inoculated plants were incubated at 28°C with full humidity, and the disease lesions were observed at 5 dpi. To observe the infection process, conidia suspension (1 × 10^5^ conidia ml^-1^) was inoculated onto the lower barley epidermis, and then incubated in a dark chamber with full humidity at 28°C. Infection process was observed at different times after inoculation under a microscope (Nikon Ni90, Japan).

### Quantitative Real-Time PCR Analysis

To detect gene expression levels at different developmental stages, samples were harvested to extract total RNA using TRIzol (Invitrogen, United States). Total RNAs were subsequently used to prepare the cDNA templates. Samples of mycelia were harvested from cultures incubated in liquid CM for 48 h. Samples of the germ tubes and appressoria were harvested form hydrophobic plastic surface at 3 and 12 hpi (2 × 10^5^ conidia ml^-1^). Samples of infection hyphae were harvested by tearing down the lower barley epidermis at 18, 24, and 42 h after inoculation with conidia suspension (2 × 10^5^ conidia ml^-1^). The qRT-PCR was performed on the ABI 7500 real-time PCR system (Applied Biosystems, United States) by using SYBR Green PCR Master Mix (Takara, Dalian, China) as manufacture’s instruction.

### Phenotypic Characterization of Mutants

Conidium germination and appressorium formation were observed on a hydrophobic coverslip. Drops of conidial suspension (1 × 10^5^ conidia ml^-1^) were inoculated onto a coverslip and incubated in a moistened chamber at 28°C. Germ tubes and appressoria formation ratios were calculated by a microscope with at least 100 conidia per replicate, and three replicates were used for per experiment.

Appressorium turgor was determined by cytorrhysis assay. Drops of conidial suspension (1 × 10^5^ conidia ml^-1^) were inoculated onto a coverslip and incubated in a moistened chamber at 28°C for 24 h. Then the cover slides were immerse in different concentrations of PEG8000 (25, 30, 35, and 40%, wt/vol) for 15 min, and then washed by distilled water. Appressoria collapse ratios were subsequently calculated under a microscope with at least 100 conidia per replicate, and three replicates were used for per experiment.

### Staining Assays

For glycogen and lipid droplets staining, conidia suspension (1 × 10^5^ conidia ml^-1^) were inoculated at different times on hydrophobic coverslip, and were then applied to staining solution (60 ml mL^-1^ KI and 10 mg mL^-1^ I_2_) (Thines et al., 2000), or Nile Red solution (Sigma-Aldrich, United States) (50 mM Tris/maleate buffer, pH 7.5, with 20 mg mL^-1^ polyvinylpyrrolidone and 2.5 μg ml^-1^ Nile Red) (Greenspan et al., 1985) for 10 min, respectively. The stained germinating conidia and appressoria were observed and photographed under an epifluorescence microscope (Nikon Ni90, Japan).

For CFW staining, mycelia were harvested from CM medium and stained with 10 μg ml^-1^ CFW (Sigma-Aldrich, United States) for 10 min in the dark, then rinsed twice with PBS buffer and observed under the microscope (Nikon Ni90, Japan). For conidial cell staining, the conidia were harvested from strains incubated for 5 days on OTA plates.

For DAB staining assay, epidermis of barley leaves infected by strains at 36 hpi were stained with 1 mg/ml DAB (Sigma-Aldrich, United States) solution (pH 3.8) for 8 h, and de-stained with an ethanol/acetic acid solution (ethanol/acetic acid; 94:4) for 1 h, then observed with an epifluorescence microscope (Nikon Ni90, Japan).

### Transmission Electron Microscopy (TEM)

Conidia and appressoria formed at 12 hpi on barley leaves were processed for transmission electron microscopy (TEM), with processing and imaging of the TEM samples performed as described previously (Soundararajan et al., 2004).

### Pulldown Assay

To purify MoUbp14-interacting proteins, a strain expressing eGFP-MoUbp14 under the constitutive promoter RP27 was obtained. Mycelia of this strain was harvested from liquid CM incubated for 48 h and ground into powder for extracting total proteins in lysis buffer [50 mM Tris–HCl (pH 7.4), 150 mM NaCl, 1 mM EDTA, and 1% Triton X-100] containing 10 μg/ml each of leupeptin and pepstatin A, and 1 mM PMSF. Resulting mixture was centrifuged at 4°C for 30 min at 15,000 rpm. The supernatant was incubated with anti-GFP affinity resins (Sigma-Aldrich, United States) and washed by several times. Then the proteins were eluted with 0.1% Rapigest as described (Zhou et al., 2012). The elution proteins were subjected to trypsin digestion and liquid chromatography tandem mass spectrometry (LC-MS/MS) as described (Zhou et al., 2012). The resulting MS/MS data were used to search against the *M. oryzae* protein database at NCBI. Two biological replicates were used for this assay.

### Western Blotting

The 3 × Flag fused *MoFBP1* or *MoPCK1* construct was constructed. Both constructs were transformed into the wild type or Δ*Moubp14* mutant, respectively. Subsequent transformants were performed with Western blot analysis with an anti-Flag primary antibody (1:5000, Sigma, United States) and an anti-rabbit horseradish peroxidase secondary antibody (1:10000, Sigma, United States). Results were visualized with the ECL detection system (Amersham Biosciences, United Kingdom). To test catabolite degradation of MoFbp1 and MoPck1, the wild type and the Δ*Moubp14* mutant strains expressing Fbp1:3 × Flag or Pck1: 3 × Flag were incubated firstly in CM medium for 42 h, and then transferred the mycelia into the liquid medium with NaAc as the sole carbon source for 12 h, and then added glucose into the medium for incubating for 2 h. Total ubiquitination levels of the wild type, the Δ*Moubp14* mutant and the complementation strain cUBP14 were detected by an anti-ubiquitin antibody (Abcam, United Kingdom).

### Statistical Analysis

For all of the biological analyses, means and SE were calculated from three independent replicates. Significant differences are derived from *t*-test with *P* < 0.01 or *P* < 0.05.

## Results

### Characterization of the *M. oryzae* Gene *MoUBP14*

The MoUbp14 (MGG_08270) was identified via a search of the *M. oryzae* genome database (Ensembl Fungi^1^) by using the *S. cerevisiae* Ubp14 protein as a query. The MoUbp14 is predicted to encode a protein of 787 amino acids with a putative RING-type zinc finger domain at its N-terminus and a ubiquitin specific protease (USP) domain at its C-terminus (Supplementary Figure S1A). Phylogenetic tree analysis by using MEGA version 5.10 demonstrated the Ubp14 protein is well conserved among eukaryotes. Among the analyzed organisms, *Neurospora crassa* (XP_960037.1) and *Fusarium graminearum* (XP_011318590.1) Ubp14 are the closest match to MoUbp14 (Supplementary Figure S1B). Multiple sequence alignment was also performed to evaluate the conservation of the Ubp14 protein among eukaryotes. The MoUbp14 protein shares a 72% positive amino acid identity with that of *N. crassa*, 71% with *F. graminearum*, 36% with *A. thaliana*, 34% with *Oryzae sativa*, 34% with *S. cerevisiae*, and 37% with *Homo sapiens* (Supplementary Figure S2) at the protein level with more than 95% query coverage.

### Expression of MoUbp14 During Different Developmental Stages

To evaluate potential roles of *MoUBP14* in *M. oryzae*, the transcription profile was examined by quantitative real-time PCR (qRT-PCR). *MoUBP14* was highly expressed during mycelial growth, appressorium formation and the late invasive growth at 48 h after inoculation (hpi), while was severely decreased during conidial germination and the early invasive growth at 18 and 24 hpi (Supplementary Figure S3). This result suggested that the expression of *MoUBP14* gene is fine tuned for development and infection processes.

To determine the roles of *MoUBP14*, a gene replacement construct was obtained to perform a homologous recombination strategy-mediated disruption (Supplementary Figure S4A). This construct was then transformed into the wild-type strain P131. Two independent Δ*ubp14* null mutants with similar phenotypes were obtained and confirmed by Southern blot analysis (Supplementary Figure S4B). One of the null mutants, ubp14KO, was randomly selected for further analysis. To confirm the phenotypic defects of the mutants were resulted from the disruption of *MoUBP14*, complementation transformants were also generated by transforming the native promoter drived *MoUBP14* into the Δ*ubp14* null mutant. All of the complementation strains was recovered in all the phenotypic defects, and one of which, termed cUBP14, was randomly selected for further study.

### Deletion of *MoUBP14* Affects Fungal Vegetative Growth and Conidial Formation

**FIGURE 1:**
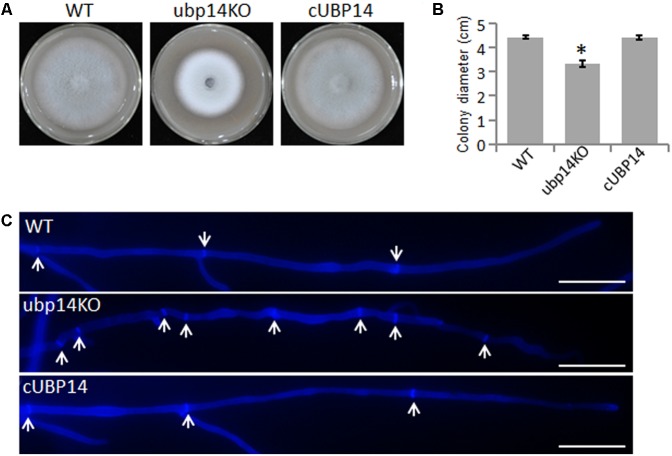
Deletion of *MoUBP14* affects vegetative growth. **(A)** The Δ*Moubp14* mutant is reduced in colony growth. The wild-type strain P131 (WT), *MoUBP14* deletion mutant (ubp14KO), and complemented strain (cUBP14) were cultured on oatmeal tomato agar (OTA) plates at 28°C for 5 days. **(B)** Colony diameters of P131, ubp14KO, and cUBP14. Significant differences compared with WT are indicated by asterisks (*P* < 0.05). **(C)** Hyphal tips of P131, ubp14KO, and cUBP14 were stained with Calcofluor White. The cell septa are indicated by white arrows. Bar, 20 μm.

To investigate whether the function of *MoUBP14* is associated with vegetative growth in *M. oryzae*, we observed colony morphology and size on oatmeal agar plate (OTA). The growth diameter of Δ*Moubp14* was significantly reduced compared with that of the wild type, and the colony was evidently whiter than the wild type (**Figures 1A,B**). When the Δ*Moubp14* mycelia was stained with Calcofluor White (CFW), we found the average lengths of apical hyphal cells were significantly reduced, and the location of septa were abnormal, compared with the wild-type (**Figure 1C**). These results indicated that *MoUBP14* is required for fungal vegetative growth and mycelial pigmentation.

**FIGURE 2:**
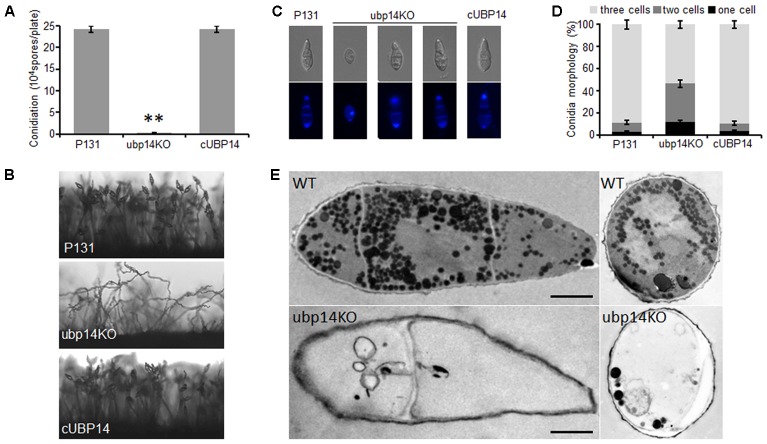
Disruption of *MoUBP14* shows abnormal conidial morphology and reduced conidial production. **(A)** Conidiation of of P131, ubp14KO, and cUBP14. Conidia were harvested from strains growing on OTA plates (Φ = 6 cm). Means and standard errors were calculated from three independent experiments (^∗∗^*P* < 0.01, *n* > 100). **(B)** Conidiophore development was observed under light microscopy. **(C)** Microscopy observation of conidial morphology. Conidial septa of P131, ubp14KO, and cUBP14 were stained with the Calcofluor White. Conidia of each strain were counted under a microscope in three independent experiments (*n* > 100). **(D)** Percentage of different conidial morphology. **(E)** TEM observation to the conidia of P131 and ubp14KO. Bar, 5 μm.

As conidia is very important for spread of the rice blast fungus, we next assessed the role of *MoUBP14* in conidiation. Conidiation measurements showed that the Δ*Moubp14* mutant produced only around 1.5% conidia of that produced by the wild type and the complementation strain (**Figure 2A**). Microscopic observation found that, compared with conidiophores with dense conidia in the wild-type and complementation strain, the Δ*Moubp14* mutant formed sparse conidia on the conidiophores after 10 days of continuous exposure to light (**Figure 2B**). Moreover, we found that conidia of the Δ*Moubp14* mutant were abmormal in conidial cell number. For the wild-type strain, more than 80% of the conidia were three-celled. While for the Δ*Moubp14* mutant, only 53.5% of the conidia were three-celled and 47.5% of the conidia were two-celled (with one septum) or one-celled (without septum). These defects were recovered in the complementation strain cUBP14 (**Figures 2C,D**). Further TEM observation demonstrated that a large number of contents, which were supposed to be glycogen, lipid body, etc., can be observed in conidia of the wild type, but only a few contents were found in most conidia of the Δ*Moubp14* mutant (**Figure 2E**). These results indicated that deletion of *MoUBP14* affects conidiation, conidiophore formation, conidia morphology and conidial deposit formation.

### Deletion of *MoUBP14* Led to Loss of Virulence

**FIGURE 3:**
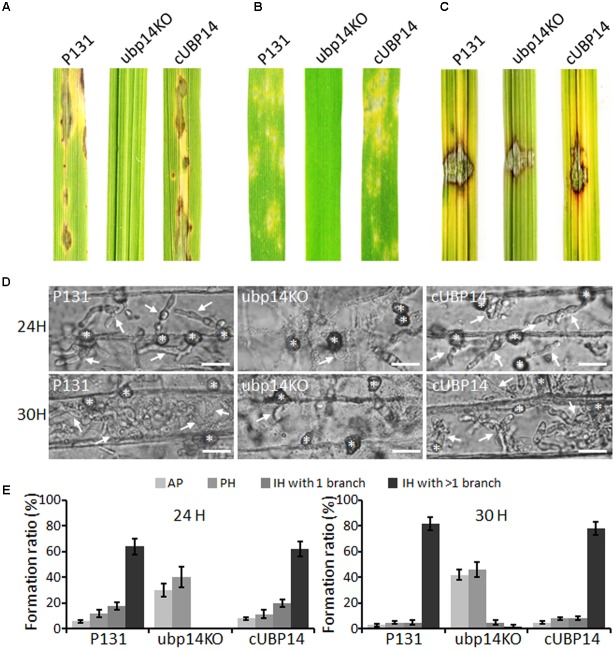
Pathogenicity assays. **(A)** Spray assay for disease development on rice seedlings. **(B)** Spray assay for disease development on barley leaves. For **(A,B)**, conidial suspension (5 × 10^4^ ml^-1^) of indicated strains was sprayed onto leaves of susceptible rice and barley and incubated for 5 days. **(C)** Pathogenicity test on abladed rice leaces. Hyphal agar plugs (5 mm in diameter) were placed onto rice leaves with treatment of wounds and incubated for 4 days. **(D)** Penetration and infection hyphae of P131, ubp14KO, and cUBP14 were examined at 24 and 30 hpi. Appressoria were indicated by asterisks, and infection hyphas were indicated by arrows. Bar, 20 μm. **(E)** Percentages of appressoria (AP), primary infection hyphae (PH), IH with 1 branch and more than 1 branch at 24 and 30 hpi, respectively.

To investigate whether the deletion of *MoUBP14* could affect pathogenicity, susceptible seedlings (*O. sativa* cv. LTH) at fifth leaf stage were inoculated by spraying conidia suspension. Compared with the wild type and complementation strains which caused numerous typical necrotic lesions, the Δ*Moubp14* mutant was completely non-pathogenic, (**Figure 3A**). A similar result can be also observed on the barley leaves infected by the same strains mentioned above (**Figure 3B**).

When inoculating the mycelial agar plugs onto the wounded rice leaves, the wild type and complementation strains can spread well and cause disease symptoms, but the Δ*Moubp14* mutant spread much slower (**Figure 3C**). This result indicated that deletion of *MoUBP14* affects host colonization. We also compared major infection steps of the Δ*Moubp14* mutant with those of the wild-type strain by observing conidia adhesion, conidia germination, appressoria formation, and host penetration and invasive growth on barley leaves as shown in below.

### *MoUBP14* Disruption Influences Appressorium Maturation

**FIGURE 4:**
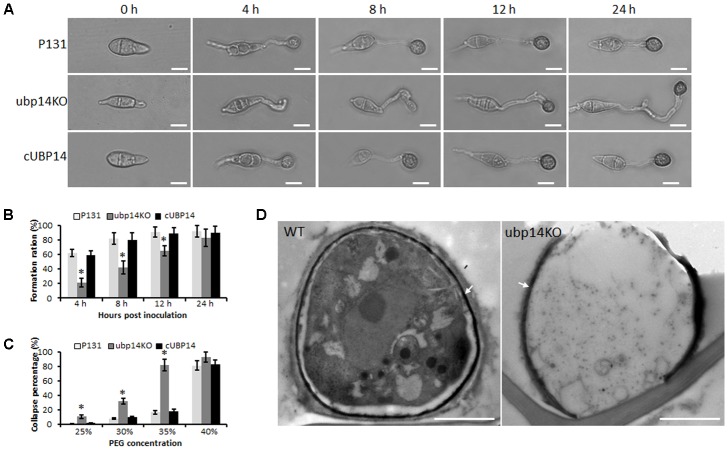
*MoUBP14* affects functions of appressorium. **(A)** Appressorium formation assay. Conidia were incubated on hydrophobicsurfaces and observed at different time points. Bar, 10 μm. **(B)** Appressorium formation rates at different time. Asterisks representsignificant differences (*P* < 0.05, *n* > 100). **(C)** Cytorrhysis assay for appressorium turgor pressure. Drops of conidial suspension (1 × 10^5^ conidia ml^-1^) were placed on the hydrophobic surface of coverslips and treated with indicated concentration of PEG8000 at 24 hpi. **(D)** Transmission electron microscopy (TEM) observation of the appressorium. Melanin layer was indicated by arrows.

To determine conidia germination and appressorium formation ability of the Δ*Moubp4* mutant, we observed the germination process. Conidia harvested from 10-day-old OMA culture plates were inoculated on hydrophobic coverslips. Nearly 85% conidia of Δ*Moubp14* can form appressoria at 24 hpi, close to that of the wild type and cUBP14. However, the germination and appressorium formation processes of the mutant were evidently delayed (**Figures 4A,B**). At 4 hpi, compared with the wild type, most of the Δ*Moubp14* conidia formed long germ tubes, only 21% of Δ*Moubp14* conidia formed appressoria, and most of which were immature. At 12 hpi, only 65% of Δ*Moubp14* conidia formed appressoria, compared with 91% of the wild type (**Figure 4B**). In order to determine whether the Δ*Moubp14* mutant formed dysfunctional appressorium, we carried out the cytorrhysis assay. When treated with different concentrations of PEG8000, appressoria formed by the Δ*Moubp14* mutant were collapsed more easily than those of the wild type and the complementation strain (**Figure 4C**), suggesting turgor pressure in the mutant was significantly reduced. TEM observation also showed that the content of the Δ*Moubp14* mutant was severely reduced, and the melanin layer was light-colored than the wild type, suggesting it formed a looser melanin layer (**Figure 4D**). Taken together, these results demonstrated that disruption of *MoUBP14* affects conidium germination and appressorium maturation during infection of *M. oryzae*.

### Appressorial Penetration and Invasive Growth Are Blocked in the Δ*Moubp14* Mutant

To further reveal why deletion of the *MoUBP14* resulted in loss of pathogenicity, we observed infection process in barley epidermal cells. We found that appressoria formed by the Δ*Moubp14* mutant were severely blocked in penetration and invasive growth in host cell (**Figures 3D,E**). At 24 hpi, more than 90% of the wild-type appressoria penetrated into the plant cells and 64% of them developed branched infection hyphae (IH). In contrast, seldom Δ*Moubp14* appressoria formed primary IH. At 30 hpi, 82% of the wild-type IH formed branched IH, whereas it was only 7% in the Δ*Moubp14* mutant (**Figures 3D,E**). The wild type and cUBP14 strains can well penetrate and colonize in the invaded host cells (**Figures 3D,E**). These results indicate that deletion of *MoUBP14* significantly affects appressorium-mediated penetration and invasive growth in *M. oryzae*.

### Deletion of *MoUBP14* Affects Stress Response

**FIGURE 5:**
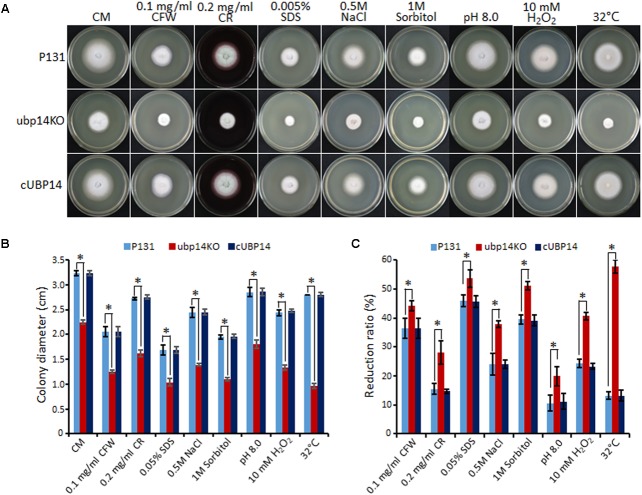
Deletion mutants of *MoUBP14* is sensitive to different stresses. **(A)** Colony morphorlogy of P131, ubp14KO, and cUBP14 on CM plates supplemented with different indicated stress agents. The colonies were photographed at 5 dpi. **(B)** Colony growth of P131, ubp14KO, and cUBP14 on CM plates supplemented with different indicated stress agents. Means and standard errors were calculated from three independent replicates. Significant differences are indicated by asterisks (*P* < 0.01). **(C)** Statistical analysis of growth reduction rates of colony growth under different stresses. Means and standard errors were calculated from three independent replicates. Significant differences were indicated by asterisks (*P* < 0.05).

Next, we evaluated the effect of *MoUBP14* disruption on stress tolerance in *M. oryzae*. The results revealed that the Δ*Moubp14* mutant was more sensitive to a series of stresses, including the cell wall perturbing reagents [0.1 mg/ml Calcofluor White (CFW), 0.2 mg/ml Congo Red (CR) and 0.005% Sodium dodecyl sulfate (SDS)], the osmotic stress (0.5 M NaCl and 1 M Sorbitol), alkaline pH (pH 8.0), oxidative stress (10 mM H_2_O_2_), and high temperature (32°C) (**Figures 5A–C**). Notably, the mutant exhibited severe sensitivity to high temperature (32°C), but at the same time, the wild type and complementation strain were slightly affected. These data indicated that *MoUBP14* is required for response to kinds of stresses.

### *MoUBP14* Is Relevant to Carbon Source Utilization

**FIGURE 6:**
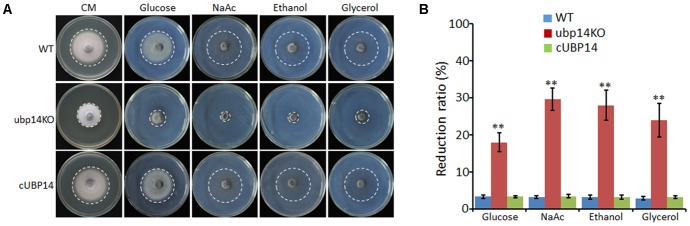
Growth tests on different carbon sources. **(A)** P131, ubp14KO, and cUBP14 cultured on CM or minimal medium agar containing 50 mM glucose, or sodium acetate, ethanol, or glycerol for 120 h at 28°C. **(B)** Statistical analysis of growth reduction rates of colony growth on different carbon sources compared to growth on CM plates. Means and standard errors were calculated from three independent replicates. Significant differences were indicated by asterisks (*P* < 0.05).

We also tested whether the disruption of *MoUBP14* can affect carbon source utilization. The wild-type, Δ*Moubp14* mutant and complementation strains were cultured on MM plates supplemented with each of glucose, NaAc, ethanol and glycerol as the sole carbon source. After 5 days culture at 28°C, colony diameter of the Δ*Moubp14* mutant was significantly reduced when grown on all of these conditions compared with itself growth on the CM plates. The mutant exhibited reduction of 39, 59, 56 and 48% in glucose, NaAc, ethanol, and glycerol conditions, respectively. In contrast, the reduction rates of the wild-type and the complementation strain were only around 6% in these carbon sources compared with themselves in the CM condition (**Figures 6A,B**). These demonstrated that the carbon sources utilization ability of the Δ*Moubp14* mutant was significantly reduced, suggested that *MoUBP14* is also relevant to carbon source utilization.

### Deletion Mutant of *MoUBP14* Is Defective in Glycogen and Lipid Metabolism

**FIGURE 7:**
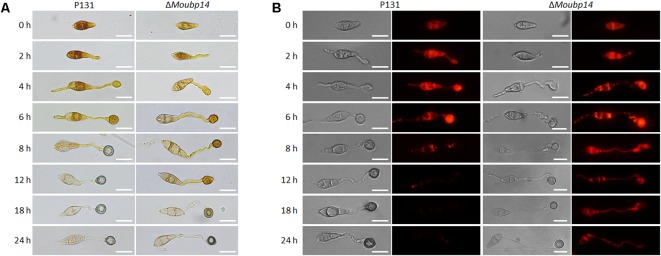
MoUbp14 contributes to glycogen and lipid translocation and degradation. **(A)** P131 and the Δ*Moubp14* mutant were stained with iodine solution at different time points and yellowish-brown glycogen deposits became visible and observed by microscopy. Bars = 20 μm. **(B)** Visualization of lipid droplets during development of appressorium. Lipid bodies of samples were stained by using of Nile Red. Bar, 20 μm.

Since the conidial storage provides nutrient utilization during the appressorium-mediated infection, we next detected the cellular distribution of glycogen and lipid. I_2_/KI solution was used to stain the glycogen. In wild type, the glycogen was abundant in conidia, along with the appressorium formation, the glycogen was translocated from conidia to nascent appressoria, and rapidly reduced in conidia and appressoria after 8 h. Then the glycogen was nearly disappeared in the mature appressoria after 12 h. In the Δ*Moubp14* mutant, glycogen was disappeared slowly in the conidia and appressoria, and noticable amount of glycogen can be still detected at 18 h, indicating the glycogen was not well utilized in the appressoria (**Figure 7A**). We also detected the lipid droplets utilization during appressorium formation by using Nile Red staining. In wild type, lipid droplets were also abundant in conidia, and rapidly transported to the incipient appressorium for degradation, and nearly diappeared at 12 h. In the Δ*Moubp14* mutant, degradation of the lipid droplets was slower than that of the wild type, and a large amount of lipid droplets can be still detected at 24 h in the conidia and germ tubes (**Figure 7B**). These results indicated deletion of *MoUBP14* affects glycogen and lipid metabolism, which is important for conidia storage utilization and functional appressorium formation in *M. oryzae*.

### The Δ*Moubp14* Mutant Led to Accumulation of Host Reactive Oxygen Species (ROS)

For the Δ*Moubp14* mutant was blocked in host cell growth, we explored whether the Δ*Moubp14* mutant resulted in host ROS accumulation. We detected ROS accumulation in the barley epidermis cells by staining with 3,3′-diaminobenzidine (DAB) at 30 hpi. Most of the barley epidermis cells infected by the Δ*Moubp14* mutant were intensely stained by DAB, which were detected with abundant reddish brown precipitates around the appressoria in the infected cells, while the host cells infected by the wild-type strain were not well stained (Supplementary Figure S5), indicating host ROS was accumulated in Δ*Moubp14* infected cells.

### Localization of MoUbp14 During Appressorium Development and Invasive Growth

In order to investigate localization of MoUbp14, the *eGFP:MoUBP14* fusion construct was obtained and transformed into the Δ*Moubp14* mutant, and the subsequent transformants were recovered in the development and pathogenicity. One of these transformants, termed Ubp14-GFP, was used for observing the subcellular localization. Conidial suspension of Ubp14-GFP was dropped on the hydrophobic cover glass and barley epidermis for fluorescence microscopic observation. GFP signals were highly expressed and detected in cytoplasm at different development stages (Supplementary Figure S6), suggesting *MoUBP14* is required in all development and infection processes of *M. oryzae*.

### MoUbp14 Disruption Resulted in Accumulation of Free Polyubiquitin Chains

**FIGURE 8:**
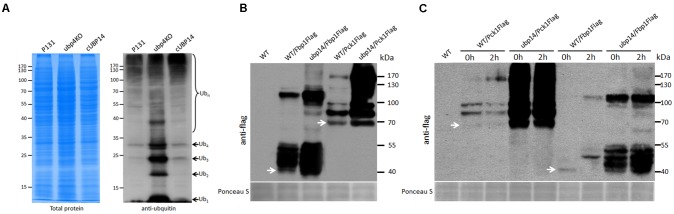
MoUbp14 is responsible for deubiquitination of MoFbp1 and MoPck1. **(A)** *MoUBP1*4 deletion mutant increased in total protein ubiquitination level. Total lysates of P131 and the Δ*Moubp14* mutant were subjected to SDS-PAGE and immunoblotted using anti-ubiquitin antibodies to detect total Ub levels. **(B)** Ubiquitination levels of MoFbp1 and MoPck1 are significantly increased in the Δ*Moubp14* mutant. MoFBP1:Flag and MoPCK1:Flag fusion constructs were transformed into P131 and the Δ*Moubp14* mutant, respectively. Corresponding transformants were analyzed by western blotting with anti-Flag antibody. Staining of total protein with Ponceau S confirmed equal loading. **(C)** MoUbp14 is required for MoFbp1 and MoPck1 degradation in short time starved condition. 12 h-starved wild type and the Δ*Moubp14* mutant strains, respectively, expressing MoFBP1:Flag (or MoPCK1:Flag) were shifted to glucose for 2 h. Total extracts were subjected to SDS-PAGE and immunoblotted using anti-Flag antibodies. Staining of total protein with Ponceau S confirmed equal loading.

In *S. cerevisiae*, Ubp14 protein functions as a deubiquitinating enzyme involved in removing ubiquitin from protein substrates (D’Andrea and Pellman, 1998). Thus, we compared the total ubiquitination levels of the wild type, the Δ*Moubp14* mutant and the complementation strain cUBP14. Total proteins were extracted from mycelia incubated in liquid CM medium. Western blot analysis was performed and ubiquitination levels were detected with an anti-ubiquitin antibody. Compared with that of the wild type and the complementation strain, the total ubiquitination level of the Δ*Moubp14* mutant was evidently increased. Notably, the monoubiquitin and polyubiquitin bands of Ub_1_, Ub_2_, Ub_3_, and Ub_4_ were massively accumulated in the Δ*Moubp14* mutant (**Figure 8A**), indicating that loss of *MoUBP14* blocked general protein degradation and chain turnover, which is consistent with previous observation in *S. cerevisiae* (Eisele et al., 2006).

### Pulldown Assay Identifies Proteins Linked to MoUbp14

We also analyzed proteins that were pulled down by Ubp14-GFP in the affinity purification analysis for identifying potential Ubp14 substrates. After filtering the background interacting proteins using the wild-type strain expressing GFP protein, a total of 31 proteins linked to Ubp14:GFP were identified in two experimental replicates (Supplementary Table S1). Interestingly, some proteins involved in carbohydrate metabolism, stress response, and two E3 ligases ptr1 and hulA, were identified as the Ubp14-interacting proteins (Supplementary Table S1), which is consistent with the roles of Ubp14 in development, nutrient utilization, stress response, and pathogenesis in *M. oryzae*.

### MoUbp14 Is Required for Degradation of Fbp1 and Pck1

Among the MoUbp14 interacting proteins, MoFbp1 and MoPck1 are two key rate-limiting enzymes in gluconeogenesis. To validate whether MoUbp14 is required for degradation of MoFbp1 and MoPck1, the constructs expressing the Fbp1:Flag and Pck1:Flag fusion proteins were introduced into the wild-type strain and the Δ*Moubp14* mutant, respectively. Total proteins from the subsequent strains were purified and immuno-blotted with anti-Flag antibody. As expected, beside Fbp1:Flag protein itself, larger bands can be detected in proteins extracted from both of the wild type and the Δ*Moubp14* mutant expressing Fbp1:Flag, however, more protein bands with massive products can be detected in the Δ*Moubp14* mutant (**Figure 8B**). Similar phenomenon can be also found for the MoPck1 protein (**Figure 8B**). These results suggested that in the Δ*Moubp14* mutant, ubiquitination levels of MoFbp1 and MoPck1 could be elevated.

In *S. cerevisiae*, when glucose is added into cells grown for around 10 h on potassium acetate as the non-fermentable carbon source, Fbp1 suffers rapid catabolite degradation by the ubiquitin proteasome pathway (UPS) (Hung et al., 2004). In order to clarify whether MoFbp1 and MoPck1 are degraded by the UPS pathway, we incubated the wild type and the Δ*Moubp14* mutant expressing Fbp1:Flag or Pck1:Flag firstly in CM medium for 42 h, and then transferred the mycelia into the liquid medium with NaAc as the sole carbon source for 12 h, and subsequently add glucose into the medium for incubating for 2 h. Total proteins from the samples were extracted and immuno-blotted by anti-Flag antibody. For both of the wild type expressing Fbp1:Flag and Pck1:Flag, when adding glucose for 2 h, protein amounts of the target proteins were significantly reduced, but some larger bands were detected or increased in amounts, compared with that of 0 h. In contrast, for the Δ*Moubp14* mutant expressing Fbp1:Flag or Pck1:Flag, protein amounts of the target proteins were significantly increased, as well as many larger bands (**Figure 8C**). This suggested that in the Δ*Moubp14* mutant, both of the MoFbp1 and MoPck1 can’t suffer rapid catabolite degradation, perhaps by the result of failing in ubiquitin recycle. These results also suggested when the glucose was supplied to *M. oryzae* grown in low glucose media for a short period of time (∼12 h), both of the MoFbp1 and MoPck1 are degraded. This process could require MoUbp14.

## Discussion

Post-translational modification by ubiquitin is essential for regulation of protein abundance and function. Previous studies have revealed importance of the ubiquitin system in the model plant pathogenic fungus *M. oryzae* (McCafferty and Talbot, 1998; Oh et al., 2012; Prakash et al., 2016; Shi et al., 2016). However, little has been addressed on the deubiquitination process. In this study, we set out to elucidate the importance of the de-ubiquitinating enzyme gene *MoUBP14* in *M. oryzae*. Our results show that deletion of *MoUBP14* resulted in vegetative growth, sporulation, cell wall integrity, stress response, and carbon source utilization. Importantly, *MoUBP14* is also required for pathogenesis. We also found degradation of two key rate-limiting enzymes in the gluconeogenesis, Fbp1 and Pck1, could require MoUbp14.

In eukaryotic cells, during modification process of ubiquitination, the monoubiquitin is constantly required, therefore, recycling or *de novo* synthesis of monoubiquitin is important. In *S. cerevisiae* and human, orthologs of Ubp14 are reported to play general roles in disassembling free polyubiquitin chains liberated from ubiquitinated proteins (Falquet et al., 1995; Wilkinson et al., 1995). Here, similar to the results of in *S. cerevisiae* (Amerik et al., 1997), human (Dayal et al., 2009), and *Drosophila* (Kovács et al., 2015), deletion of *M. oryzae UBP14* also led to accumulation of free polyubiquitin chains (**Figure 8A**), suggesting that Ubp14 may also function as a ubiquitin recycler to help cells to supply sufficient free ubiquitins.

In *S. cerevisiae*, deletion of *UBP14* led to reduction in sporulation and increase in sensitivity to the arginine analog canavanine (CAN) (Amerik et al., 1997). In dictyostelium, deletion mutants of *UbpA* (ortholog of *UBP14*) are normal in vegetative growth, but defective in aggregation, chemotaxis, cell adhesion, and formation of the fruiting bodies (Lindsey et al., 1998). In *A. thaliana*, *AtUBP14* is essential for early embryo development (Doelling et al., 2001). Comprehensive functional analysis of the DUB family in the model human pathogenic fungi *Cryptococcus neoformans* has also performed. None of the 19 putative *C. neoformans* DUB genes were essential and most DUB deletion mutants were normal in growth under standard conditions (Liu O.W. et al., 2008; Fang et al., 2012). Among these DUBs, Ubp5, Doa4, Ubp13, and Ubp14 influenced pigment production (Fang et al., 2012). Ubp5 is likely the major deubiquitinating enzyme for stress responses in *C. neoformans*, for the ubp5Δ mutant is severely reduced in virulence, capsule formation, melanization, sporulation, and was more sensitive to distinct stressors (Fang et al., 2012). In our study, we found that the MoUbp14 is one of the most important DUBs in *M. oryzae*, it is also interesting to determine functions of other DUBs proteins in *M. oryzae*.

We reasoned that the loss of virulence in the Δ*Moubp14* mutant should be due to several cellular mechanisms. Firstly, the penetration of the mutant was significantly blocked, which should be at least the result of reduced appressorial turgor pressure. Cytorrhysis analysis indicated the Δ*Moubp14* mutant is lack of turgor pressure (**Figure 4C**). The appressorium builds up enormous turgor pressure by accumulating glycerol, which is retained by the melanin layer. TEM picture demonstrated that the Δ*Moubp14* mutant formed much looser melanin layer compared with the wild type (**Figure 4D**). A defect in cell wall integrity of Δ*Moubp14* also contributes to a failure in sustaining high turgor pressure. Insufficient turgor generation in appressoria of Δ*Moubp14* is also evidently resulted from defect in utilization of glycogen and lipid storage (**Figure 7**). Secondly, the invasive growth of the Δ*Moubp14* mutant in host cells was also arrested, because the mutant retarded in invasive growth on wounded host leaves (**Figures 3D,E**). This defect could be partly due to reduction of abilities in stress adaptation and nutrient utilization (**Figures 5**, **6**). The Δ*Moubp14* mutant was sensitive to distinct stresses, including oxidative stress. According to the DAB staining assay, we found massive ROS accumulated in the Δ*Moubp14* mutant infected host cells (Supplementary Figure S5). On the other hand, the Δ*Moubp14* mutant was also reduced in utilization of different carbon sources, suggesting a lack of nutrient assimilation of the mutant in host cells.

Meanwhile, the Δ*Moubp14* mutant colony was much whiter than the wild type (**Figure 1A**), suggesting the mycelial melanin-related pigmentation could be affected by *MoUBP14*, which would also lead to reduction of cell wall integrity in the mutant. Hyphal tip cells of the ubp14KO mutant were shorter than the wild type (**Figure 1B**), which could contribute to slow growth in colony growth. This result suggested deletion *MoUBP14* could affect cell-cycle related phenotypes. As shown in **Figure 2E**, few contents were observed in the conidia of the ubp14KO mutant. Most of these contents may be nutrient storages, such as glycogen and lipid, which are required for functional appressorium formation (Wilson and Talbot, 2009).

Deletion of *MoUBP14* can affect resistance of *M. oryzae* to distinct stresses, including cell wall perturbing agents, salt stress, osmotic stress, oxidative stress, alkaline stress, and high temperature (**Figure 5**). In yeast, it has also been reported that the ubiquitin-proteasome system is involved in responding to various extracellular or intracellular stresses such as high temperature, oxidative damage, high salt, and antifungal drugs (Finley et al., 2012). Presumably, distinct stresses would lead to accumulation of misfolded or damaged proteins, and which should be removed by the ubiquitin-dependent degradation pathway. Our results from *MoUBP14* suggested that the deubiquitination process could also affect removing of misfolded proteins in responding to stresses. In studies of *C. neoformans*, deubiquitinating enzymes Ubp5 and Doa4 also play roles in resistance to different stresses, such as high sodium ions, 0.02% SDS and 0.1% caffeine, as well as antifungal agents (Fang et al., 2012), which is consistent with our results. The DUBs could be used to maintain ubiquitin homeostasis under stresses. Some signaling pathways, such as MAPK signaling pathway (Xu and Hamer, 1996), Ca^2+^/calcineurin signaling pathway (Lee and Lee, 1998), TOR signaling pathway (Yu et al., 2014), PacC signaling pathway (Landraud et al., 2013), and SNF1 kinase signaling pathway (Zeng et al., 2014) have been revealed to play roles in different stress responses or development in plant pathogenic fungi. Whether and how the DUBs can affect these signaling pathways is an intriguing question.

Deletion of *MoUBP14* affects carbon sources utilization of *M. oryzae* (**Figure 6**). Glycolysis and gluconeogenesis are reversible metabolic pathways, which is essential for carbon sources utilization in different organisms. Most of the glycolytic reaction steps are reversible, but phosphofructokinase and pyruvate kinase catalyze irreversible glycolytic pathway reactions, which are, respectively, catalyzed by Fbp1 and Pck1 in gluconeogenesis pathway (Entian and Barnett, 1992). The gluconeogenic pathway is sensitive to glucose. Upon glucose addition, Fbp1 and Pck1 will be subject to transcriptional repression, mRNA degradation (Yin et al., 2000) and protein degradation (Funayama et al., 1980; Mazon et al., 1982). In *S. cerevisiae*, glucose repression represents an attractive system to study the co-ordination of gene responses at transcriptional and post-transcriptional levels. When glucose is added into cells grown for 16–18 h on ethanol as the non-fermentable carbon source, Fbp1 suffers rapid catabolite degradation by the ubiquitin proteasome pathway (Hämmerle et al., 1998). This process is driven by so-called glucose induced degradation deficient (GID) genes, among which, GID6 encodes the deubiquitinating enzyme Ubp14 (Santt et al., 2008). However, specific biological function has not been assigned to the UPS-mediated degradation of Fbp1 in yeast. Here, we showed that the *M. oryzae* Ubp14 could be required for UPS-mediated degradation of Fbp1, as well as Pck1. We hypothesize that MoUbp14 can help in providing sufficient ubiquitin monomers for degradation. Consequently, the UPS-mediated degradation of MoFbp1 and MoPck1 may be involved in the fungal pathogens to coordinate gluconeogenesis and glycolysis for nutrient utilization during infection.

## Conclusion

In summary, these results suggest that the MoUbp14-mediated deubiquitination process is required for pleiotropic regulation during development and pathogenicity of *M. oryzae*.

## Author Contributions

ZW, HZ, and CL performed most of the experiments and data processing. ZW performed the Western blot analyses. X-LC and JX designed the experiments. X-LC, ZW, and JX wrote the manuscript.

## Conflict of Interest Statement

The authors declare that the research was conducted in the absence of any commercial or financial relationships that could be construed as a potential conflict of interest.

## Footnotes

1 http://fungi.ensembl.org/Magnaporthe_oryzae/Info/Index
