# Supplementary material for: A Deubiquitinating Enzyme Ubp14 Is Required for Development, Stress Response, Nutrient Utilization, and Pathogenesis of Magnaporthe oryzae
Source: Front Microbiol. 2018 Apr 18;9:769. doi: 10.3389/fmicb.2018.00769 (PMC5915541; doi:10.3389/fmicb.2018.00769)
Supplement: Supplementary file 2 [file Image_1.PDF]

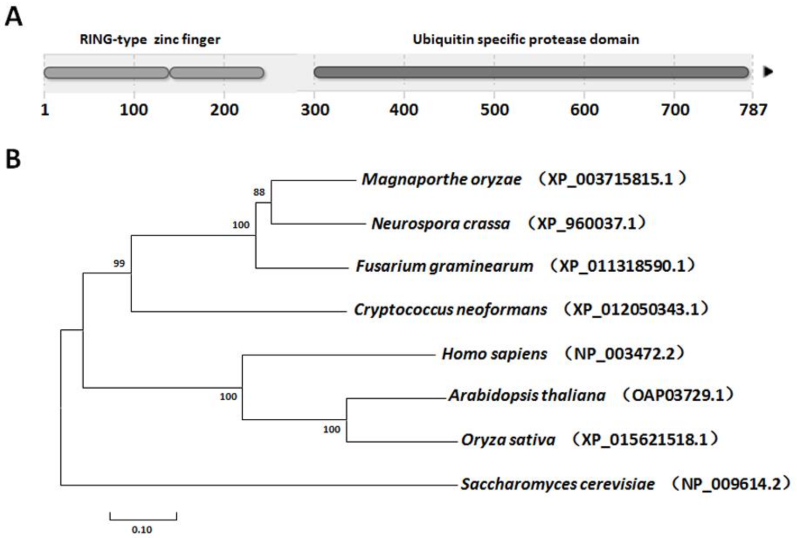

FIGURE S1 | Conserved domains and phylogenetic tree analyses of Ubp14 proteins. **(A)** MoUbp14 protein conserved domains predicted by InterProScan (http://www.ebi.ac.uk/interpro/search/sequence-search). **(B)** A neighbour-joining tree of the amino acid sequence of related proteins. Numbers at nodes represent the percentages of occurrence, and the scale bar represents the number of amino acid differences per site. GenBank accession numbers are followed by species.

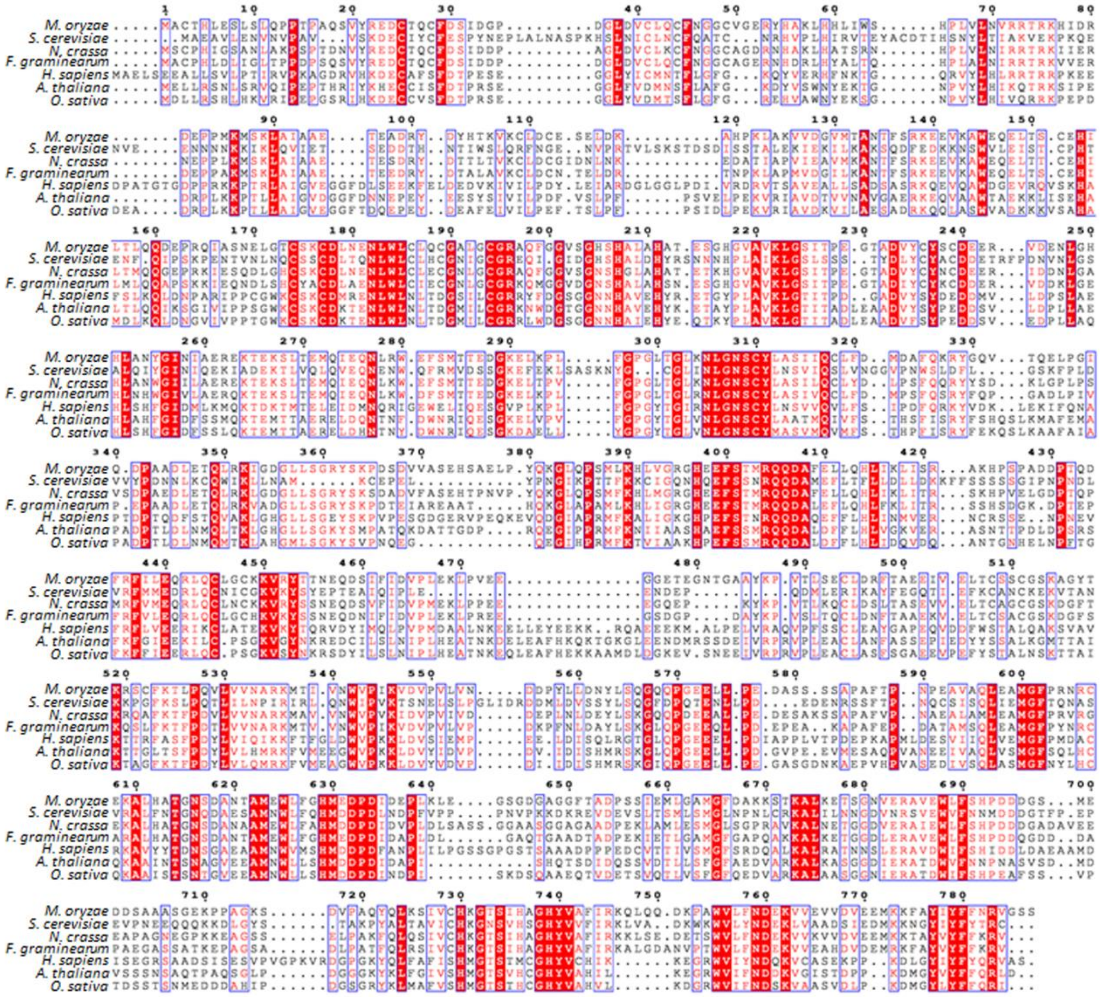

FIGURE S2 | Alignment of the amino acid sequences of Ubp14. Amino acid sequences were obtained by BLAST and aligned with CLUSTAL W (http://www.ch.embnet.org/software/ClustalW.html). Identical and similar residues are indicated by red regions and red characters, respectively. Sequences aligned were the predicted products of *M. oryzae* Ubp14 (XP_003715815.1), and Ubp14 orthologues from *S. cerevisiae* (NP_009614.2), *Neurospora crassa* (XP_960037.1), *Fusarium graminearum* (XP_011318590.1), *Homo sapiens* (NP_003472.2), *Arabidopsis thaliana* (OAP03729.1) and *Oryza sativa* (XP_015621518.1).

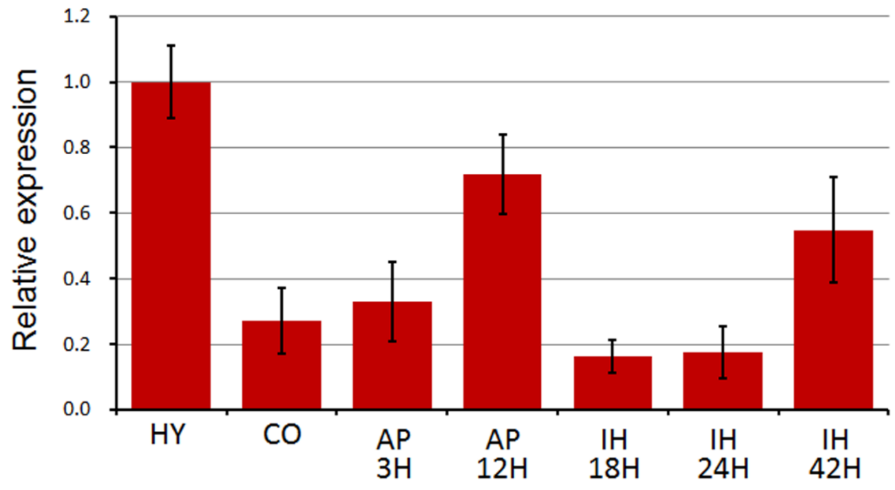

FIGURE S3 | Phase specific expression of *MoUBP14*. The phase specific expression of *MoUBP14* was quantified by quantitative real-time PCR with synthesis of cDNA from each sample including mycelia, conidia, germ tubes, appressoria and infection hyphae at indicated time points. Relative abundance was normalized by *MoTub1*. Three independent biological experiments with three replicates in each were performed. HY : Mycelial hyphae; CO: Conidia; AP : Appressoria ; IH : infection hyphae.

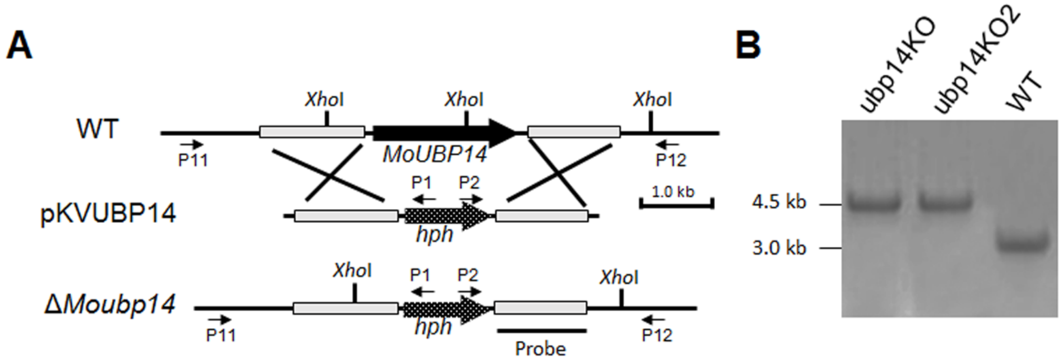

FIGURE S4 | Targeted disruption of *MoUBP14*. **(A)** Schematic diagram of the *MoUBP14* deletion strategy. *HPH* indicates the hygromycin B phosphotransferase gene cassate. *Xho*I restriction enzyme site is used in the Southern blot analysis. **(B)** Confirmation of the Δ*Moubp14* mutant by Southern blot analysis. *Xho*I-digested genomic DNAs were hybridized with the probe.

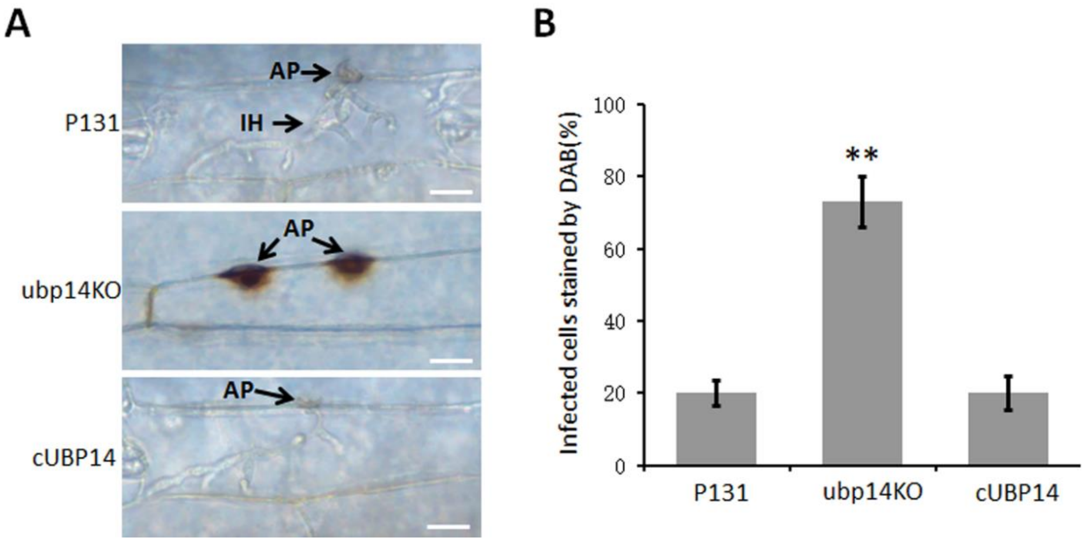

FIGURE S5 | DAB staining assay. **(A)** Barley epidermis was stained with DAB at 30 hpi and observed under a Nikon microscopy. AP, appressoria; IH, infection hyphae. Bar, 20 μm. **(B)** Percentages of DAB stained cells. Means and standard errors were calculated from three independent replicates. Significant differences were indicated by double stars ($P < 0.01$, n > 100).

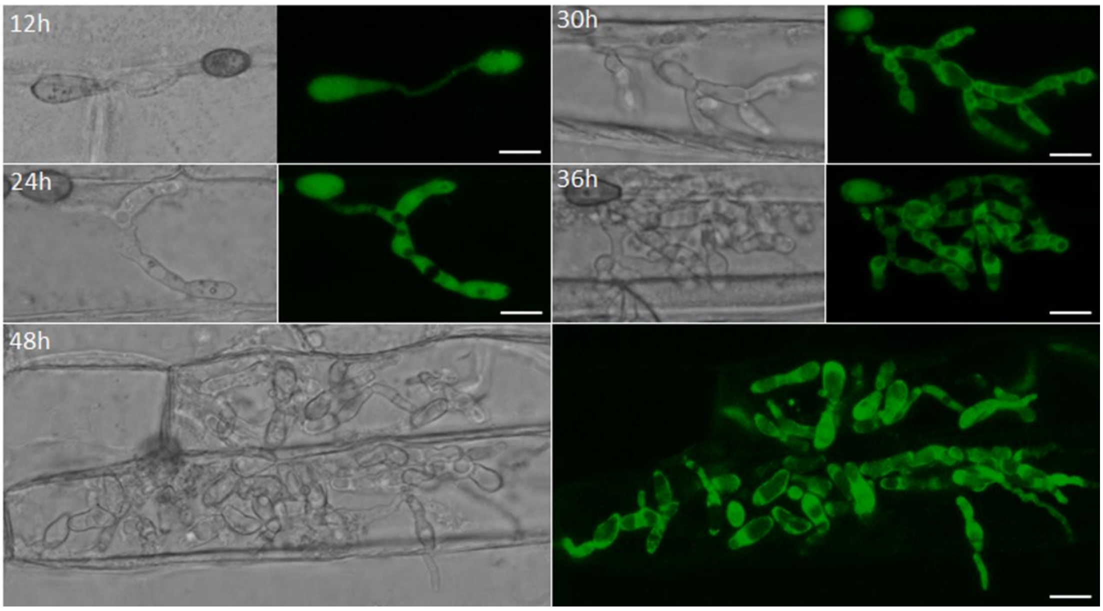

FIGURE S6 | Subcellular localization of MoUbp14. MoUbp14 is localized to the cytoplasm of the conidia in the complemented strain.The subcellular localization of MoUbp14 during appressorium and invasive growth was performed by inoculate conidia suspensions on barley epidermis and observed at different time points.
